# Molecular and metabolic traits of some Egyptian species of *Cassia* L. and *Senna* Mill (Fabaceae-Caesalpinioideae)

**DOI:** 10.1186/s12870-022-03543-7

**Published:** 2022-04-20

**Authors:** Marwa M. Eldemerdash, Ashraf S. A. El-Sayed, Hussein A. Hussein, Samir S. Teleb, Rania S. Shehata

**Affiliations:** 1 Botany and Microbiology Department, Faculty of Science, Zagazig University, Zagazig, 44519 Egypt; 2 Biology Department, Faculty of Science, Jazan University, Jazan, Saudi Arabia

**Keywords:** *Cassia*, *Senna*, Taxonomical features, ITS sequence, RAPD analysis, GC-MS profile

## Abstract

The genus *Cassia* and *Senna* have been classified under subfamily Caesalpinioideae of family Fabaceae (Leguminosae) of order Fabales. There is a scarce taxonomical studies of the genus *Cassia* and Senna inhabiting Egyptian environments, thus, the main objective of the current was to revise and authenticate the phylogenetic relationship between studied taxa of the species of the genera *Cassia* and *Senna* in Egypt using the recent tools of ITS barcoding, RAPD analysis and metabolic profiling, in comparing to the traditional taxonomical features. From the cluster analysis of the traditional 27 morphological characters, the studied taxa were categorized into two major clades with an average taxonomic distance of 4.3. The clade I include *Cassia fistula, C. renigera*, *C. javanica* L subsp. nodosa and *C. roughiia* that belongs to series Obolospermae, and *C. grandis* that belongs to series Grandes. The clade (II) includes *Senna surattensis* and *S. alata* at taxonomic level 3.6. The taxonomical description of the studied taxa was confirmed from the molecular analysis of ITS sequences and RAPD analysis. The ITS sequences of the tested plants species *C. fistula* L, *C. grandis* MD4, *C. javanica* subsp. nodosa MD7, *C. roxburghii* MD5, *C. renigera* MD5 were deposited at genbank with accession numbers MW367973, MZ960447, MW386305, MW326753 and MW32685, respectively. While, the ITS sequences of the *S. surrattensis* and *S. alata* were deposited into genbank accession # MD14 MW367670 and MD20 MW412635, respectively. Thus, from the molecular analysis, two clades were clearly separated into Clade I of *Cassia* and Clade II of *Senna*. The cluster I represented by *C. fistula*, C. *renigera*, *C. roxburghii*, and *C. javanica* sub *nodosa,* and the cluster II represented by *S. alata* and *S. surattensis.* From the PCA of RAPD, a clearly discrimination between the two Taxa was observed revealing the characteristic grouping of *Cassia* and *Senna*. The species *Senna alata* and *Senna surattensis* were grouped together, but the species of *C. renigera*, *C. javanica, C. roxburghii* and *C. grandis* was grouped on a distinct group. The separation of *Cassia* and *Senna* species into two clusters verify the segregation of the genus *Cassia* L. senso lato into two distinct genera namely *Senna* P. and *Cassia* L. The morphological, molecular traits of the studied plants were authenticated from the metabolic profiling by GC-MS analysis. Among the 23 identified metabolites, four compounds namely hexadecanoic acid, methyl ester, 9-Octadecenoic acid (Z)-ethyl ester and Vitamin E were detected with fluctuated concentrations, among *C. fistula, C. grandis*, *C. javanica* subsp. *nodosa* and *C. roxburghii.* Conclusively, the traditional morphological features, molecular barcoding using ITS sequences, RAPD analysis and metabolic traits by GC-MS analysis, authenticates the taxonomical diversity of the genus *Cassia* and *Senna*.

**Supplementary Information:**

The online version contains supplementary material available at 10.1186/s12870-022-03543-7.

## Introduction

The genus *Cassia* L. and *Senna* Mill. have been classified under subfamily Caesalpinioideae of family Fabaceae (Leguminosae) of order Fabales [1, 2]. *Cassia* and *Senna* were segregated into the three genera: *Chamaecrista* Moench., *Senna* Mill. and *Cassia* L. [3–11]. This segregation was subsequently reinforced based on ontogenetic floral development studies Tucker [12] as well as using the molecular biology tools [13]. The genus *Cassia* include only 30 species [14]. Comparing to about 400 species in *Cassia* sens. Lat. as reported by Brenan [15]. Five species of *Cassia* sens. Lat. were introduced as horticulture plants in Egypt [16]. The genus *Senna* Mill, Gard. Dict. includes around 350 species and spread over the world [3, 4, 17] among them 17 species were introduced as horticulture plants in Egypt. Species of *Cassia* and Senna are widely grown as ornamentals [18] extensively used in various parts of the world as remedies for various human ailments [19, 20]. Species of *Cassia* sens. Lat. and *Senna* are well known for their laxative and purgative uses [21, 22] antioxidant activity [23] anticancer [24] and antimicrobial activities [25, 26]. In addition, these plants were used to treat gastrointestinal disorders, some skin diseases and wound healing [27, 28]. The extensive variability in its growth habit ranging from tall trees to delicate annual herbs, numbers and size of the leaflets, form and foliar characteristics has added difficulties to taxonomists in identification of species or the intraspecific taxa for influence of habitat conditions [7]. Taxonomically, *Senna* and *Cassia* are very complex genus owing to the strong polymorphism of a number of species and the absence of intrageneric incompatibility.

Recently, various taxonomical tools have been implemented such as anatomical, cytological, serological, genetic characteristics and metabolic traits [29–31], rather than floral and vegetative character, that have been reported as important features in determining the relationships and affinities of the plants. With the development of various taxonomical tools that based on the molecular and metabolic traits, new taxonomical systems have been evolved, exploring new criteria for assessing the evolutionary status of the individual taxa in the subtribe Cassiinae. Recently, several molecular markers for demonstrating the intra, and interspecific genetic variations have been implemented for the direct comparison of the plant variation at biochemical and molecular levels [32, 33]. Random amplified polymorphic DNA (RAPD) analysis and sequences of the internal transcribed spacer (ITS) are one of the recent molecular tools for separating the particular species from their ancestors. Unlike traditional morphological features, the molecular tools especially RAPD and ITS sequences are independent on the environmental changes that gave these approaches more credibility. RAPD is a reliable predictive, rapid tool to clarify areas of maximum diversity and evaluate natural genetic diversity in plant populations [34, 35]. The sequence of ITS region is one of the most authenticated molecular markers in the evolutionary investigations at various taxonomic levels. DNA sequence is the straightforward since the nrDNA sub-units contain large numbers of copies, with numerous copies of rRNA genes within a genome, relatively homogenous, coupled with the different subunits and spacer sections [36]. The ITS area is particularly common because of the highly nucleotide replacement rate of the transcribed intervals which allows a taxonomical comparison of the highly similar divergent species [37]. The ITS region is impacted by the coordinated evolution that homogenizes the tandem copies across individuals, making ribosomal DNA accessible to phylogenetic analysis [38, 39], as well as for determining the origin of plants and their derivatives [40, 41]. Comparison of DNA sequences within the species is a powerful approach for determining the evolutionary forces acting in specific gene regions, and also for determining the relevant aspects of the evolutionary history of the species [42]. Due to the higher rate of nucleotide substitution, relative feasibility of amplification and the large available sequence data, the internal transcribed spacer (ITS) regions of the nuclear ribosomal cistron (18S-5.8S-26S) has been considered as a very successful tool for species-level discrimination across flowering plants [43–45].

Recently, the emergence of DNA sequence data allows the quantitative comparison of nucleotide polymorphism levels at species and populations and corresponding degrees of population sequence divergence [46]. In addition, metabolic profiling by GC-MS of plants is one of the major recent trends for authentication of the morphological taxonomical and molecular features of plants [47]. From literature, there is a scarce taxonomical studies of the genus *Cassia* and Senna inhabiting Egyptian environments, thus, the main objective of this study was to implement the various molecular and biochemical tools for confirming and revisiting the taxonomical identities of these plants. Thus, the objective of this study was to revise and authenticate the phylogenetic relationship between studied taxa of the species of the genera *Cassia* and Senna in Egypt using the recent tools of ITS barcoding, RAPD analysis and metabolic profiling, in comparing to the traditional morphological and taxonomical features.

## Materials and method

### Collection and identification of plant samples

Seven horticultural taxa representing the genus *Cassia* and *Senna* comprising six species and one subspecies were the subject of this study. Fresh plant material, for each one of them, was collected since April 2020 (Table 1). *Cassia fistula* L. was collected from Parks at Faculty of Science, Zagazig University, Zagazig, Egypt. *Cassia grandis* L.f. and *Senna surattensis* (Dc.) Irwin and Barneby were collected from Zohria Trial Gardens, Gezira, Giza, Egypt. *Cassia javanica* L. subsp. nodosa, *Cassia renigera* Benth. and *Cassia roxburghii* DC were collected from Orman Botanic Garden, Giza, Egypt (Table 1). The plants were obtained after permission from Zagazig University, Orman and Zohria Botanical gardens. The voucher herbarium specimens were prepared and matched for identification with the authentic ones at the Orman Botanical and Zohria Botanical garden, Giza, Egypt. The plants were identified by the official staff members of the Orman Botanical Garden (OBG), Zohria Botanical Garden (ZBG), Cairo university Botanical garden, with the identification numbers as included on Table 1.

**Table 1:** The collection data of the taxa studied of *Cassia* and their assignment into their corresponding series [4]

Taxa	ID number	Locality	longitude & latitude	Series
*Cassia fistula* L.	ZUBG-09	Parks at Faculty of Science, Zagazig University, Zagazig, Egypt.	30.5765°N 31.5041°E	*Cassia*
*Cassia grandis* L. f.	ZBG-008	Zohria Trial Gardens, Gezira, Giza, Egypt.	30.0131°N 31.2089°E	*Grandes*
*Cassia javanica* L. subsp. *nodosa* (Roxb.) K. Larsen & S. S. Larsen	OBG-109	Orman Botanic Garden, Giza, Egypt.		*Obolospermae*
*Cassia renigera* Benth.(Synonym: *Cassia javanica* L. subsp. *renigera* Benth.)	OBG-104	Orman Botanic Garden, Giza, Egypt.		*Obolospermae*
*Cassia roxburghii* DC. (Synonym: *Cassia marginata* Roxb.)	OBG-101	Orman Botanic Garden, Giza, Egypt.		*Obolospermae*
*Senna surattensis* (Dc.) Irwin&Barneby	ZBG-006	Zohria Trial Gardens, Gezira, Giza, Egypt		Subverrucosae
*Senna alata* (L.)Roxb	*CUBG-002*	Cairo University Botanical Garden	30.0444° N 31.235° E	Pictae

*ZUBG* Zagazig University Botanical Garden, *ZBG* Zohria Botanical Garden, *OBG* Orman Botanical Garden, *CUBG* Cairo University Botanical garden

### Morphological studies

Twenty-eight characters were investigated according to the reference keys for taxonomic classification of *Cassia* and *Senna* [48–50]. The morphological characters and character states were determined by examining of the living specimens, and were coded as multistate characters. Ten individuals from each plant were used for the morphological description. One individual has been used for the molecular and biochemical analyses. While one ind The data matrix was analyzed using multistate matrix. The data matrix was subjected to cluster analysis using UPGMA (Unweighted pair group method with arithmetic mean) and the phylogenetic relatedness was constructed to show the relationship among the taxa. All analyses were carried out using the program Past (Version 4.3c) [51]. The Morphological Characters descriptions were recorded in Table 2.

**Table 2:** Primer sequence of ITS and RAPD analysis

	Name	Base Pair Primers (bp)	Prime Primer Sequence (5-3)	Source
RAPID	ABI-07	10 bp	GGTGACGCAG	
	ABI-08	10 bp	GTCCACACGG	
	ABI-09	10 bp	TGGGGGACTC	
	ABI-10	10 bp	CTGCTGGGAC	
	ABI-11	10 bp	GTAGACCCGT	
	ABI-12	10 bp	CCTTGACGCA	
ITS	ITS2 2F	20 bp	ATGCGATACTTGGTGTGAAT	Chen et al., [52]
	ITS2 3R	21 bp	GACGCTTCTCCAGACTACAAT	Gao et al., [53]

### Molecular study

#### Molecular identification of the plant samples

The plant genomic DNA was extracted by CTAB lysis buffer [52]. Fresh weight of the plant tissue (0.1 g) was pulverized into fine powder in liquid nitrogen, the CTAB lysis buffer (500 μl) was added, vortex for 1 min, and centrifuged at 10000 rpm for 10 min [53]. Equal volume of chloroform was added to the supernatant, vigorously shaking, centrifuged at 10,000 rpm for 10 min, the upper layer was taken and amended with double volume of ethanol, incubated at − 20 °C for 30 min, centrifugation for 10 min to pellet the DNA. The DNA pellets were dissolved in 50 μl distilled water and stored at − 20 °C, and checked by 1.5% agarose gel, normalizing to 1 kb ladder (Cat. # PG010-55DI). The ITS primer sets were listed in Table 3. The reaction mixture contains 10 μl of 2 × PCR master mixture (i-Taq™, Cat. # 25,027), 2 μl of gDNA, 1 μl of the primers (10 pmol/μl), and completed to 20 μl with sterile distilled water. PCR amplification was performed at Thermal Cycler 006, programmed to initial denaturation 94 °C for 2 min, denaturation 94 °C for 20 s, annealing 51 °C for 30 s, extension 72 °C for 1 min for 35 cycles, with final extension 5 min at 72 °C. The PCR amplicons were analyzed by 1.5% agarose gel in 1 × TBE buffer, and sequenced by Applied Biosystem Sequencer (HiSQV Bases, Version 6.0). The obtained sequences were non-redundantly BLAST on the NCBI, and the FASTA sequences were aligned with Clustal W muscle algorithm [54].

**Table 3:** Morhological charcaters of *Cassia* and *Senna*

Character	*Cassia fistula L*	*Cassia grandis*	*Cassia renigera*	*Cassia roxburghii DC*	*Cassia javanica*	*Senna alata*	*Senna. surattensis*
1-Life Span	perennial	perennial	perennial	perennial	perennial	perennial	perennial
2-Life form	Tree	Tree	Tree	Tree	Tree	Shrub	Shrub
3-stem surfaces	glabrous	pubescent	pubescent	pubescent	pubescent	pubescent	pubescent
4-Leaf duration	deciduous	deciduous	deciduous	deciduous	deciduous	evergreen	evergreen
5-Leaflet pairs in numbers	8-14 pairs	14-20paris	16-20pairs	14-20 pairs	16-20 pairs	8-12 pairs	6-12 pairs
6-Leaflet shape	ovate	oblong	oblong	oblong	oblong	obovate-oblong	obovate-oblong
7-Leaflet margin	entire	entire	entire	entire	entire	entire	entire
8-Leaflet apex.	acute	obtuse	obtuse	obtuse	acute	obtuse	obtuse
9-Leaflet base	Obtuse	Obtuse	Obtuse	Oblique	Obtuse	Oblique	Oblique
10-Leaflet adaxial surface	glabrous	puberulent	puberulent	glabrous	puberulent	glabrous	glabrous
11-Leaflet abaxial surface	puberulent	tomentose	tomentose	puberulent	puberulent	puberulent	puberulent
12-Leaflet lenght	7.5-15 cm	5-7 cm	5-10 cm	7-10 cm	5.5-10	6-12 cm	2-5 cm
13- Leaflet width	2.5-7 cm	1-2 cm	0.4 -2 cm	1-2 cm	0,6-2 cm	3-6 cm	0.8-2 cm
14- Stipule	cauducous	cauducous	cauducous	cauducous	cauducous	Persistent	cauducous
15- Stipule shape	deltoid to ovate	deltoid to ovate	kidney	kidney	linear to lanceolate	Triangular	linear to lanceolate
16-Bract shape	ovate	Linear to lanceolate	leafy	Linear	Ovate	oblong to broadly ovate	linear to lanceolate
17- Sepals shape	ovate	ovate	ovate	ovate	ovate	oblong	ovate
18-Sepals colour	green	redish	redish	redish	redish	yellowish-green	yellowish-green
19- Petals shape	obovate	obovate	obovate	Ovate Oblong	obovate	ovate	obovate
20- Petals colour	yellow	pink	pink	pink	pink	yellow	yellow
21-pod Curvature	straight	straight	straight	straight	straight	straight	straight
22- Pod colour	Black	Dark brown	Dark brown	Dark brown	Dark brown	brown	Dark brown
23- Pod Texture	glabrous	glabrous	glabrous	glabrous	glabrous	glabrous	hairy
24- POD APEX	rounded	rounded	rounded	rounded	rounded	ACUMINATE	rounded
25- Dehiscence of pod	indehiscent	indehiscent	indehiscent	indehiscent	indehiscent	dehiscent	dehiscent
26- Seed shape	elliptic	obovate-elliptic	obovate-elliptic	elliptic	obovate-elliptic	deltoid	obovate-oblong
27- Seed color	Brown	Brown	Brown	Brown	Brown	Black	Dark brown

#### Sequence analysis

Alignment analysis of the ITS sequences were adjusted using BioEdit version 7.2.5 [55], for each sequence, length and GC contents were estimated using the Endmemo software (http://www.endmemo.com/bio/gc.php) (Table 4). The derived ITS nucleotide sequences were analyzed with MEGA version X software [56]. The sequences were manually checked and the pairwise sequence divergence between studied taxa in ITS1, 5.8S and ITS2 regions was calculated according to the Maximum Composite Likelihood (MCL) [56], verified by comparing with the sequences of other species by Basic Local Alignment Search Tool (BLAST). Positions containing gaps and missing data were eliminated from the dataset, support values of the internal branches of NJ tree were evaluated through bootstrap method (1000 replicates). The transition/transversion ratio ti/tv was estimated using the following formula R = [A*G*k1 + T*C*k2]/[(A + G)*(T + C)] with A, G, C, T as the corresponding frequencies of four nucleotides [57]. The number of nucleotide substitutions per site between sequences was estimated. The aligned sequences in the Mega files were analyzed with DnaSP software version 4.0 [58] to estimate polymorphism indices. The average of nucleotide differences (k) and the minimum number of recombination events (Rm) are also estimated. Selection neutrality was tested by both Tajima’s D [59] and Fu and Li’s D* and F* methods [60].

**Table 4:** The studied taxa, location and their geographical distribution

Scientific name	NCBI accession No.	Length bp	GC%
1-*Cassia fistula* L	MW367973	**796 bp**	**58.29**
2- *Cassia grandis*.	MZ960447	439 bp	
3-*Cassia renigera* Benth (Synonym: *Cassia javanica* L. subsp. *renigera* Benth.)	MW326851	**738 bp**	**58.53**
4-*Cassia roxburghii* DC. (Synonym: *Cassia marginata* Roxb.)	MW326753	**732 bp**	**63.25**
5- *Cassia javanica* L subsp. *nodosa* (Roxb.) K. Larsen & S. S. Larsen	MW386305	**737 bp**	**59.52**
6- *Senna. surattensis* (Burm. f) Irwin & Barneby	MW367670	**729 bp**	**60.08**
*7- Senna alata* (L.) Roxb. (Synonym: *Cassia alata* Linn.)	MW412635	**403 bp**	**59.55**

#### RAPD analysis

The molecular diversity of the studied Taxa was assessed by RAPD analysis [61]. The primer set of 20 random decamer oligonucleotides were purchased (Metabion International AG, Planegg, Germany) as listed in Table 3. The reaction mixture contains 10 μl of 2 × PCR master mixture, 2 μl of gDNA, 1 μl of each primers (10 pmol/μl), and completed to 20 μl with sterile distilled water. PCR amplification was performed at Thermal Cycler 006, programmed to initial denaturation 94 °C for 2 min, denaturation 94 °C for 20 s, annealing 51 °C for 30 s, extension 72 °C for 1 min for 35 cycles, with final extension 5 min at 72 °C. The PCR amplicons were analyzed by agarose gel in 1 × TBE buffer (Cat# AM9864). For each primer in RAPD PCR, the number of polymorphic and monomorphic bands was determined. Bands clearly visible in at least one genotype were scored (1) for present, and (0) for the absent and entered into a data matrix. Fragment size was estimated by interpolation from the migration distance of marker fragments. Percentage of Polymorphism Information Content (PIC) was calculated by applying the formula given by [62, 63], Where fi is the frequency of the i^th^ allele, and the summation extends over alleles. Then PIC values were used to calculate marker index (MI). In addition, principal component analysis (PCA) scatter diagram was constructed based on Dice coefficient genetic similarity matrix by using PAST, ver. 4.02 software [51].

$$\mathrm{PIC}=\underset{\mathrm{i}=1}{\overset{\mathrm{n}}{1-\sum f{\mathrm{i}}^2}}$$

Where f i is the frequency of the i^th^ alleles and the summation extends over n alleles.

#### Numerical analysis

Data analysis was performed using the PAST, ver. 4.02 software [51]. Jaccard’s similarity coefficients were used to generate a dendrogram using Unweighted Pair Group Method with Arithmetic Average (UPGMA) [64] and relationships between the samples were represented. In addition, principal component analysis (PCA) scatter diagram was constructed based on Dice coefficient [65] using SIMQUAL module of the program. The hierarchical clustering analysis was generated using (UPGMA).

### GC-MS metabolic profiling

#### Preparation of plant leaves extracts

Harvested healthy fresh leaves from the collected specimens, for each taxon, were shade dried in the laboratory for 2 weeks and crushed to a dry powder using a kitchen blender. The powdered leaves (10 g) were extracted by cold maceration [47, 66, 67] with 50 ml methanol (1:5 w/v) for 72 h at room temperature in tightly sealed conical flasks. Each extract was filtered using muslin cloth, the filtrates were collected and centrifuged. The supernatant was collected and the solvent was evaporated to 5 ml final volume, and then stored in tightly sealed dark vials at 4 °C till use.

#### GC-MS analysis of the compounds from the leaves extracts

The chemical constituents of each extract was determined with the Trace GC1310-ISQ mass spectrometer (Thermo Scientific, Austin, TX, USA) using a direct capillary column TG-5MS (30 m × 0.25 mm × 0.25 μm film thickness). The column oven temperature was initially hold at 50 °C, then increased by 5 °C/min to 230 °C for 2 min, and increased to the final temperature 290 °C by 30 °C/min and hold for 2 min. The injector and MS transfer line temperatures were kept at 250 °C, and 260 °C respectively. Helium was used as a carrier gas at constant flow rate of 1 ml/min. The solvent delay was 3 min and diluted samples of 1 μl were injected automatically using Autosampler AS1300 coupled with GC in the split mode. EI mass spectra were collected at 70 eV ionization voltages over the range of m/z 40-1000 in full scan mode. The ion source temperature was set at 200 °C. The chemical identity of the components was identified by comparison of their retention times and mass spectra with those of WILEY 09 and NIST 11 mass spectral databases.

## Results and discussions

### Morphological analysis

**Fig. 1:**
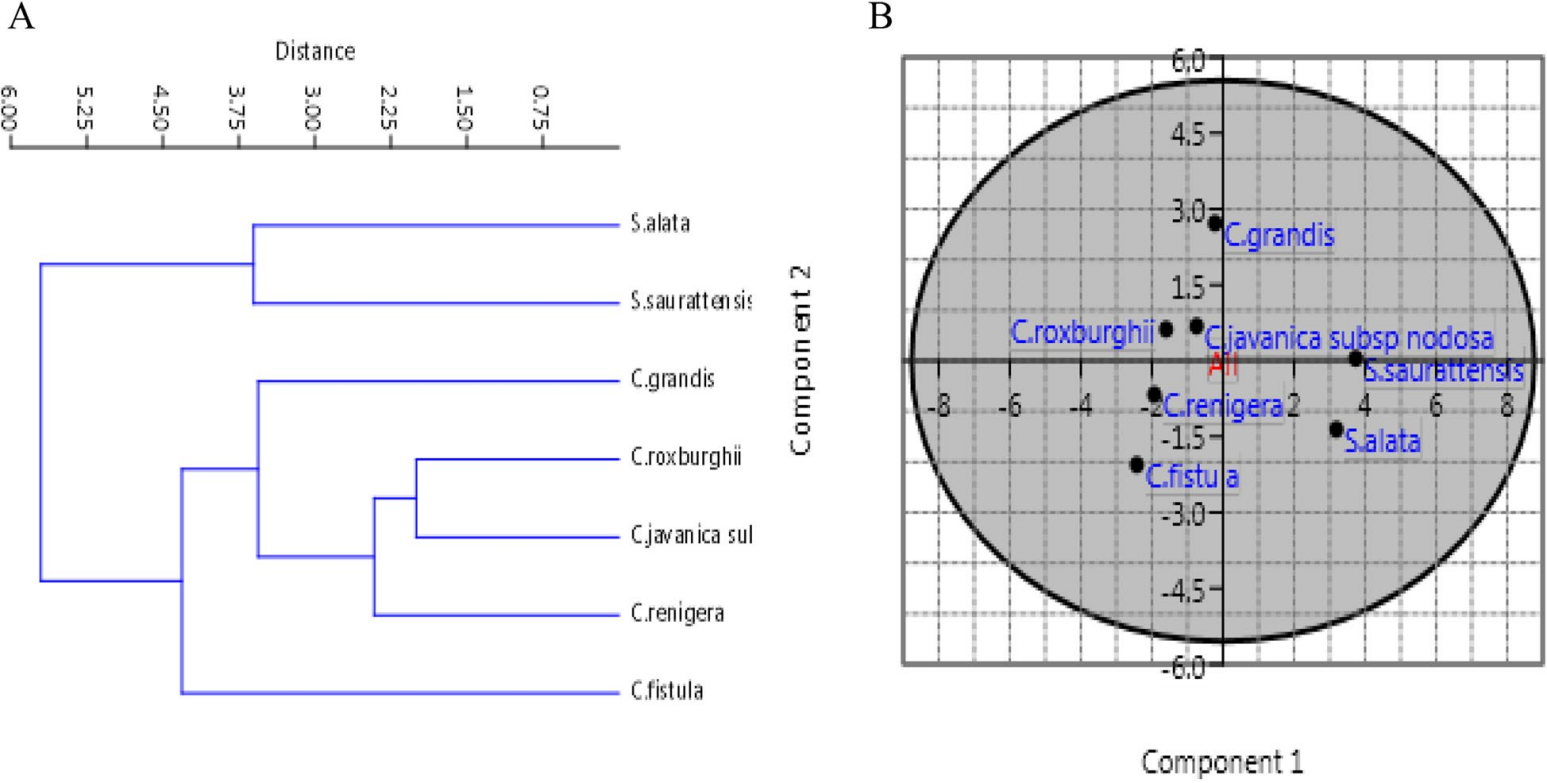
UPGMA analysis (**A**) an PCA analysis (**B**) of Cassia and Senna species based on the morphological features

Six species and one subsp. of *Cassia* and *Senna* were collected from different localities; Zagazig, Giza and Cairo Egypt, with different longitudes and latitudes as summarized in Table 1. Based on the traditional taxonomical criteria which approximately represented by 27 characters (Table S1), the different species of *Cassia* and *Senna* series were represented. *Cassia fistula* L. and *C. grandis* belonging to *Cassia* and Grandis series, respectively, were identified. While three species of namely; *Cassia javanica*, *C. renigera* and *C. roxburghii* were described to belongs to the series Obolospermae. As well as based on the above 27 taxonomical feature, the species *Senna surattensis* and *S. alata* were described to be belonging to Subverrucosae and Pictae, respectively [3, 4]. The universal morphological features of *Cassia* and *Senna* plants were described in Table 2. The UPGMA dendrogram clusters generated from the 27 morphological characters (Fig. 1) classified all studied taxa into two major clades and have an average taxonomic distance of about 4.3. The first clade was divided into three groups; group one includes *Cassia fistula* which belongs to series *Cassia* at taxonomic level 4.4. *Cassia renigera*, *Cassia javanica* L subsp. nodosa and *Cassia roughiia* which belongs to Series Obolospermae separated at in one group at a distance level at 2 taxonomic distance level third group includes *Cassia grandis* at taxonomic level 3.06. Clade (II) includes *Senna surattensis* and *Senna alata* at taxonomic level 3.6. The leaf morphological variations of all the plant species were shown (Table 3), that strongly agrees with the series level by [3, 4]. There is a considerable degree of genetic variety in several Cassiinae species derived via investigation by molecular markers, as coincide with other morphological markers [68–70].

### PCA analysis

The PCA analysis reflects the distribution and incidence of the different morphological traits of the experimented plants, by plotting the PC1 and PC2. From the PCA scatter plot, a clearly discrimination between the two Taxa was observed revealing the characteristic assemblage of *Cassia* and *Senna*. The species of *S. alata* and *S. surattensis* were grouped together; the species of *C. renigera*, *C. javanica, C. roxburghii* and *C. grandis* was separated on a distinct group (Fig. 1). The interspecific genetic divergence refers to the genetic variation within the species, with the clear separation of the two genera *Cassia* and *Senna* as coincident with the criteria of morphological and molecular features. The separation of *Cassia* and *Senna* species into two clusters prove the segregation of the genus *Cassia* L. senso lato into two distinct genera; *Senna* P. Mill., and *Cassia* L. senso stricto [3, 4].

### Molecular analyses of the experimental plants

#### Internal transcribed spacers (ITS) analysis

**Fig. 2:**
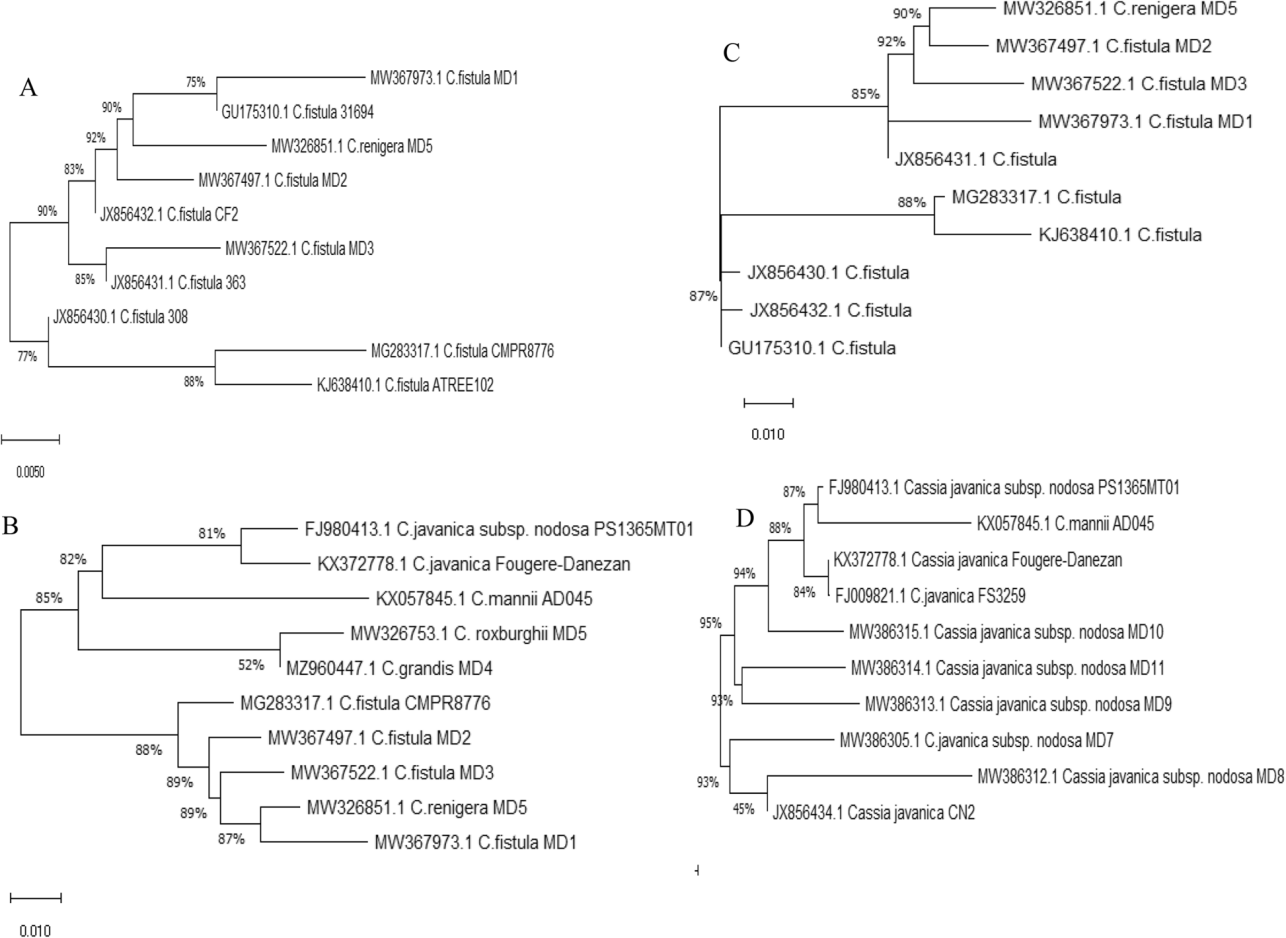
Molecular Phylogenetic analysis of the *Cassia* species based the ITS sequences for *Cassia fistula* (**A**), *Cassia grandis* (**B**), *Cassia renigera* (**C**) and *Cassia javanica* subsp nodosa (**D**)

The sequence of ITS region has been utilized as universal molecular phylogenetic marker for plant differentiation between various species [71]. The sequence of this has been frequently authenticated for differentiation of the interdependent and intra-specific interactions of plants [72, 73]. The genomic DNA of the plants were used as PCR primer for amplification of the ITS regions. From the PCR amplicons (Fig. 2), the size of DNA was around 600-700 bp, the products were sequenced and BLAST searched non-redundantly on NCBI database. According to the Neighbor-Joining (NJ) method, the studied taxa have been separated into two different clusters segregated the subtribe Cassiinae. The first cluster includes all *Cassia* species, while the second one includes all species of *Senna*. The first cluster (I) divided into two sub cluster, the first one include *Cassia grandis* MD4 MZ960447 that clearly separated, which belongs to Series Grandis while the other group includes and *C. javanica* subsp. nodosa MD7 MW386305. *C. roxburghii* MD5 MW326753, *C. renigera* MD5 MW32685, which belongs to Series oblospermaea the infra-generic arrangement of species in *Cassia* and *Senna* was in agreement with [3, 4], with an obvious deviations regarding to intrageneric relationships *C. fistula* MD1 MW3679973 in the same group. This might be due to the selection of a small number of species from such a large taxon for the present investigation and amplification of a small portion of the entire genome [74]. A significant difference in chromosome size, morphology and condensing behavior among members of the controversial subtribe Cassieae (Cassia, Chamaecrista and Senna) was revealed on the tribe to suggesting the heterogeneous group from the karyological view [3, 4, 75] (Irwin and Barneby 1982,1981, Souza and Benko-Iseppon, 2004). The second cluster includes *S. surrattensis* MD14 MW367670 and *S. alata* MD20 MW412635.

**Fig. 3:**
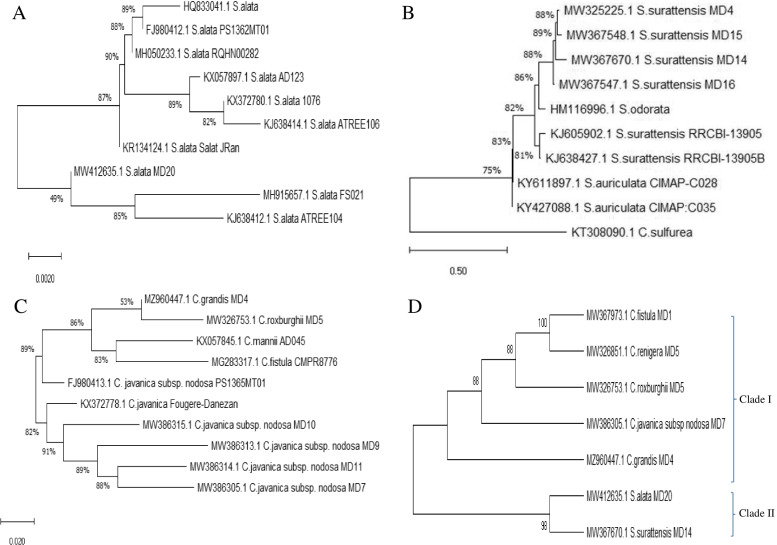
Molecular Phylogenetic analysis of the *Cassia* and *Senna* species based the ITS sequences of *S. surrattensis* (**A**), *S. alata* (**B**), *C. roxburghi* (**C**). The phylogenetic relatedness of the *Cassia* and *Senna*

From the alignment profile, *Cassia fistula* displayed 99% similarity with various species of *Cassia fistula*, has been deposited on gene bank under accession number MW367973. *Cassia fistula* displayed a 99% similarity with *Cassia fistula* JX856431.1, JX85643.1, JX856430.1, MG283317.1, KJ638410.1, GU175310.1, MW326851.1 with E value zero and query coverage 96%. The ITS sequences of tested species of *C. grandis* MD4*, C. roxburghii* MD5, *C. renigera* MD5, *C. javanica* subsp. *nodosa* MD7 were deposited to the genbank with accession numbers MZ960447, MW326753, MW326851 and MW386305, respectively (Fig. 3). From the alignment profile, *Cassia grandis* MD4 MZ960447 displayed 99% similarity with different species of *C. fistula* MG283317.1, MW3674971.1, MW367522.1, MW32685.1, MW367973.1, and MW326753.1 with E value zero and query coverage 99%. From inspection of database deposited sequences there is no ITS sequences of *C. grandis* on the genbank, so, this is the first report confirming the taxonomical identity of *C. grandis,* inhabiting the Egyptian environment. *Cassia roxburghii* MD5 MW326753.1 displayed a 98% similarity with *C. fistula* MG283317, *C. javanica* FJ980413.1, KX372778.1, MW386315.1, MW386314.1 and MW386305.1 with E value zero and query coverage 98%. Obviously, there is no ITS sequences of *C. roxburghii* deposited on the database, so, the similarity has been conducted non-redundantly towards the database deposited sequences. *Senna surattensis* MD14 MW367670 displayed 99% similarity with *S. surattensis* KY611897.1, KY427088.1, KJ638427.1, JY427088.1, MW367547.1, MW367670.1 and MW325225.1 with E value zero and query coverage a 90%. Based on the ITS sequence, the phylogenetic analysis of the experimented *Cassia* and *Senna* (Fig. 3), two phylogenetic clades, in which *Cassia* belongs to Clade I, and *Senna* belongs Clade II. From the molecular relatedness, the two species of experimented *Senna* were apparently distinct from the tested *Cassia* plants, ensuring the difference on the conserved sequences of ITS regions, or might be due to evolutionary. These molecular discriminations being consistent with the recent taxonomical traits based on the morphological features. Traditional taxonomical features such as macro-morphological and micromorphological characters are restricted by the deficiency of clear criteria for character selection, lacking the uniform standard and credible coding data, so causing somewhat misidentification. Therefore, confirmation of the morphological taxonomical features with the recent molecular tools such as DNA barcoding and molecular markers are one the most recent trends for confirming the traditional morphological features, and exploring the phylogenetic relationships between closely related taxa and their effect on their morphological identification. From the traditional taxonomical traits, the subtribe Cassinnae contains the genus *Cassia* and *Senna* [3, 4]. From the molecular analysis, two clades were clearly separated into Clade I of Cassia and Clade II of *Senna*, thus, conclusively the molecular analysis and morphological features being consistent. The taxonomical features of the subtribe Cassinnae were described in details (Table S1), as result from the UPGMA dendrogram clustering algorithm using 27 morphological traits that indicated a strong relationship between seven taxa in two clusters (Fig. 1). The cluster I represented by *C. fistula*, C. *renigera*, *C. roxburghii*, and *C. javanica* sub *nodosa,* and the cluster II represented by *S. alata* and *S. surattensis.*

The number of nucleotides substitution from sequences of the ITS sequences from the tested *Cassia* and *Senna* species were represented in Table 5. Three parameters and seven nucleotide sequences were used in the study, including 1st + 2nd + 3rd + nonecoding codon positions [56]. For each pair of sequences all unclear locations were deleted (pairwise deletion option). The final dataset had a total of 837 locations. Analysis of distance matrix shows high level of genetic distance (1.470) was observed between MW367670, *S. surattensis* MD14, and MZ960447, *C. grandis* MD4. Low level of genetic diversity (0.0292) between MW367973 *C. fistula* MD1 and MW326851 *C. renigera* MD5 was observed.

**Table 5:** Maximum composite likelihood estimate of the pattern of nucleotide substitution

	A	T	C	G
**A**	–	*4.23*	*6.67*	**11.75**
**T**	*4.69*	–	**21.58**	*6.9*
**C**	*4.69*	**13.7**	–	*6.9*
**G**	**7.99**	*4.23*	*6.67*	–

Each entry shows the probability of substitution (r) from one base (row) to another base (column) [1]. For simplicity, the sum of r values is made equal to 100. Rates of different transitional substitutions are shown in bold and those of transversionsal substitutions are shown in *italics*. The nucleotide frequencies are 20.86% (A), 18.83% (T/U), 29.65% (C), and 30.67% (G). The transition/ transversion rate ratios are *k*_*1*_ = 1.704 (purines) and *k*_*2*_ = 3.238 (pyrimidines). The overall transition/ transversion bias is *R* = 1.16, where *R* = [A*G*k_*1*_ + T*C*k_*2*_]/[(A + G)*(T + C)]. This analysis involved 7 nucleotide sequences. Codon positions included were 1st + 2nd + 3rd + Noncoding. All ambiguous positions were removed for each sequence pair (pairwise deletion option). There were a total of 837 positions in the final dataset. Evolutionary analyses were conducted in MEGA X [2]

#### Length variation, GC content, nucleotide composition, and mutational events of ITS

The obtained sequences demonstrating the differences in the GC content of the investigated species (Table 4). The sizes of ITS sequences were varied from 403 bp to 796 bp in *Senna alata* MD20 and *Cassia fistula* MD1, respectively. The GC contents were ranged between 58.29 and 63.25% in *C. renigera*, *C. roxburghii*. The transition/transversion rate ratios are *k*_*1*_ = 1.704 (purines) and *k*_*2*_ = 3.238 (pyrimidines). The overall transition/transversion bias is *R* = 1.16, where *R* = [A*G*k_*1*_ + T*C*k_*2*_]/[(A + G)*(T + C)]. There were 7 nucleotide sequences in this study. Position 1st + 2nd + 3rd + noncoding was added for the codon. Every sequence pair of unclear places has been deleted (pairwise deletion option). The completed dataset has a total of 837 places, with the replacements (Table 5). The transitions on the intergenic spacer ITS are more common than transversion, there are 20.86% (A), 18.83% (T/U), 29.65% (C), and 30.67% of the nuclear frequencies (G) of species *Cassia* and *Senna*. According to these findings, the fluctuation in the composition of the ITS nucleotide alignment into the 837 character matrix indicated that there were 212 conserved sites, 582 variables comprising 159 informative sites, and 395 singleton loci (Table 6). The frequency of nucleotides composition was 20.86, 18.83, 29.65 and 30.67% accordingly for A, T, C and G, that being consistent with that reported for *Quercus* spp. [76]. Similar studies were reported for Wheat (597 - 605 bp) and Barley (595 - 598 bp) [77]. The whole ITS variation spanned between 650 and 850 bp in the Asteraceae family, the average nucleotide frequency was A (25%), T (24%), C (26%), and G (25%) Average GC content was 51% and AT 49% [78]. The mean length of *Ficus carica* of ITS was 697.5 bp and its composition was 19.7% (18.6%) [37]. The *Chili* ITS1-5.8S-ITS2 analyses indicated nuclear frequencies of 18.85% (A), 17.56% (T), 33.95% (C), guanine (G) and 29.64% (A) and average length of 620 bp of thymine (T), respectively [79] The ITS region in *Coniferales, Cycadales, Ginkgoales* and *Gnetales* was ranged between 575 and 700 bp in angiosperms and between the species of 975 and 3125 bp in the range [80] *Phoenix dactylifera* with the mean ITS level of genetic diversity is 2% in the overall data set. These findings are comparable to those observed in *Quercus suber* and *Q. Trojana* [81], *Glycine max* [82] and *Tylosema esculentum* [83]. The transition/ transversion ratio R of 1.16 registered in the entire ITS region that is lower than that the entire ITS region in Tunisian cultivars of date palm (ti/tv = 4.375) [84], Asteraceae (ti/tv = 1.43) [78], *Capsicum* sp. (ti/tv = 3.746) [79].

**Table 6:** Nucleotide diversity, sequence polymorphism based on ribosomal DNA of Cassia and Senna species

Parameter	Frequency
**m**	**7**
**n**	**837**
**s**	**582**
**C**	**212**
**ps**	**0.695341**
**Θ**	**0.283812**
**π**	**0.228936**
**Eta**	**310**
**Tajima’s D**	**−1.24870**
Fu and Li′s D*	**−0.98694**
Fu and Li′s F*	**−1.16035**
**Fu’s Fs statistic**	**1.474**

This analysis involved 7 nucleotide sequences. Codon positions included were 1st + 2nd + 3rd + Noncoding. All ambiguous positions were removed for each sequence pair (pairwise deletion option). There were a total of 837 positions in the final dataset. Evolutionary analyses were conducted in MEGA X [2]

*Abbreviations*: *m* Number of sequences, *n* Total number of sites, *S* Number of segregating sites, *C* Conserved sites, *ps* S/n, *Θ* ps/a1, *π* nucleotide diversity, and Total number of mutations, Eta Eta

#### Selective neutrality tests

Comparative analysis of the DNA sequences within and between species is one of the most powerful approaches for determining the evolutionary domains in specific gene regions, and for determining the relevant aspects of the evolutionary history within the species [42, 85]. The pattern of plant diversity was analyzed from the neutrality test of the experimental plants (Table 6). The ribosomal nuclear DNA sequence was notably different from the neutral balancing model. Selective neutrality for the detected variations was tested by both Tajima [59, 60] methods to examine the null hypothesis. Tajima D is − 1.24870. Fu and Li′s D* test statistic: − 0.98694 Statistical significance: *P* > 0.10 Fu and Li′s F* test statistic: − 1.16035. Statistical significance: Not significant, *P* > 0.10. Similar studies were reported for the Tunisian fig cultivars (*Ficus carica* L.; Moracea) and recorded higher and significant negative values for these parameter: Fu’s Fs = − 8.668 for ITS1, Fu’s Fs = − 7.093 for 5.8S gene, Fu’s Fs = − 4.40 for ITS2 and Fu’s Fs = − 5.88, for the intergenic spacer of ribosomal DNA (ITS) [37]. However, positive and not significant values of D*: 0.92037; *P* > 0.10, F*: 0.86550; *P* > 0.10 were recorded in the ITS region of Tunisian date palm cultivars (*Phoenix dactylifera* L) [84]. The neutrality statistics in D Tajima, Fu, Li and Fu support neutrality across the ribosomal DNA region (ITS). The average number of pairwise nucleotide differences, k: 99.667. Nucleotide diversity, Pi: 0.25887, Theta (per site) from Eta: 0.32865, Theta (per sequence) from Eta: 126.5306.

#### Random amplified polymorphic DNA (RAPD) analysis

**Fig. 4:**
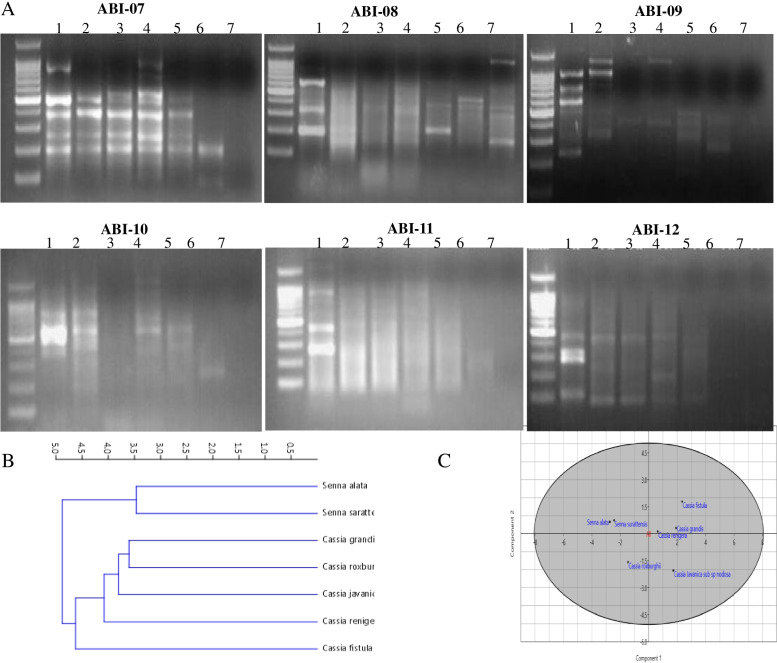
UPGMA analysis of based on the RAPD Markers of seven different taxa of *Cassia* & *Senna* generated. **A** RPAD profile analysis of the experimented plants with the different primers, **B** PCA analysis of the tested plants based on the RAPD profile

The molecular similarity of the tested plants *Cassia* and *Senna* was verified from RAPD analyses. RAPD analysis has been recognized as one of the authentic molecular tools for confirmation of the traditional taxonomical features [13]. The genomic DNA of the plants was used as PCR template with a set of ten-mer oligonucleotide primers applied to the studied species of *Cassia* and Senna (Table 3). PCR was conducted with random six primer resulted reproducible profiles in the studied species of *Cassia* and *Senna*. The PCR amplicons for each primer for the tested plants were shown in Fig. 4. A total 130 bands were scored from PCR amplification of genomic DNA with all the species. In RAPD profiling, a total of 47 clear and reproducible bands was produced, of which 46 bands were polymorphic and only one band was monomorphic which generated by ABI-08 primer. The obtained bands were ranged in size from 100 to 1200 bp. The largest amplicon 1200 bp was amplified by the primers ABI-09, ABI-10 ABI-11, and the shortest amplicons 100 bp by ABI-12. Maximum numbers of 9 amplification products were obtained with primer ABI-09 followed by 8 products with primer ABI-07, ABI08, ABI-10, and ABI-12. Minimum numbers of RAPD products were generated with primers ABI-11. The polymorphic information contents (PIC) ranged from 0.33 to 0.45 with an average of 0.37. The highest RAPD marker index (MI) (**4.05**) was found in primer ABI-09 and the lowest (**2.31**) in ABI-08 (Table 7)*.* Jaccard’s similarity index was ranged from 0.575 to 0.068, as shown in Table 8. The highest similarity value (0.575) was recorded between *C. grandis, C. javanica* subsp. *nodosa* and the lowest similarity value (0.068) between *C. grandis, Senna surattensis* and *C. javanica* subsp. *nodosa* and *S. surattensis.* The phylogenetic relatedness of RAPD analysis was constructed using UPGMA and the hierarchical clustering using PAST 4.3e as shown in Fig. 4. The RAPD analysis of the current genera was consistent with the morphological, conventional taxonomical features of the subtribe of Cassinnae as adopted by [3, 4]. From the results, the seven taxa of subtribe Cassinae were separated into two clusters for the genus *Cassia* and the genus *Senna*. The UPGMA phenogram generated from the hierarchical clustering analysis of RAPD marker illustrated that *C. fistula* is delimited as a different identity at distance coefficient of 4.5 from the remainder taxa which are clustered together in one group (Fig. 4). Within this group *C. renigera* is delimited as a different identity at a distance coefficient of 4.0 as revealed from the UPGMA clustering*. Cassia javanica* subsp. *nodosa* was delimited at a distance coefficient of about 3.8, while both of *C. roxburghii* and *C. grandis* were clustered together at a distance of about 0.74 (Fig. 4).

**Table 7:** Analysis of polymorphism among species of *Cassia* and *Senna* obtained with 6 random primers

Primers name	Primer Sequence (5′-3′)	Size Range of Amplified Product (bp)	Total no. of amplicon	Total number of bands	No of Monomorphic bands	Number of Polymorphic bands	(%) Polymorphism	PIC = 2 × fi × (1-fi)	MI=PIC × Number of Polymorphic bands
ABI-07	GGTGACGCAG	1000-100	27	8	0	8	100	0.35	2.8
ABI-08	GTCCACACGG	1000-180	27	8	1	7	87.5	0.33	2.31
ABI-09	TGGGGGACTC	1200-250	24	9	0	9	100	0.45	4.05
ABI-10	CTGCTGGGAC	1200-200	21	8	0	8	100	0.40	3.2
ABI-11	GTAGACCCGT	1200-200	15	6	0	6	100	0.39	2.34
ABI-12	CCTTGACGCA	900-100	16	8	0	8	100	0.34	2.72
Total			130	47		46			
Average								0.37

**Table 8:** Jaccard’s coeffcient of similarity indices between 7 species of Cassia and senna as revealed from RAPD using 6 primer

Taxa	***Cassia fistula***	***Cassia grandis***	***Cassia renigera***	***Cassia roxburghii*** DC	***Cassia javanica*** L.. subsp. ***nodosa***	***Senna alata***	***Senna. surattensis***
***Cassia fistula***	**100.00**						
***Cassia grandis***	**0.512**	**1**					
***Cassia renigera***	**0.297**	**0.518**	**1**				
***Cassia roxburghii*DC**	**0.512**	**0.575**	**0.464**	**1**			
***Cassia javanica*L.. subsp.*nodosa***	**0.388**	**0.482**	**0.333**	**0.387**	**1**		
***Senna alata***	**0.105**	**0.166**	**0.142**	**0.093**	**0.181**	**1**	
***Senna. surattensis***	**0.117**	**0.068**	**0.111**	**0.068**	**0.1**	**0.076**	**1**

The PCA analysis reflects the strength of the RAPD markers to classify the examined Taxa by plotting the PC1 and PC2. From the PCA scatter plot, a clearly discrimination between the two Taxa was observed revealing the characteristic grouping of *Cassia* and *Senna*. In addition, the species *Senna alata* and *Senna surattensis* were grouped together, but the species of *C. renigera*, *C. javanica, C. roxburghii* and *C. grandis* was grouped on a distinct group (Fig. 4C). The interspecific genetic divergence refers to the genetic variation within the species, with clear separation of the two genera *Cassia* and *Senna*, as revealed from the coincidence criteria of morphological and molecular features [3, 4]. The separation of *Cassia* and *Senna* species into two different clusters verify the segregation of the genus *Cassia* L. senso lato into two distinct genera namely *Senna* P. Mill., and *Cassia* L. senso stricto. The consistence of morphological and molecular taxonomical features of the subtribe Cassinae for grouping into two genera *Cassia* and *Senna* has been reported [86, 87]. Based on vegetative and reproductive characteristics, *Cassia fistula* was assigned to series *Cassia* a while *C. renigera*, *C. javanica* subsp. *nodosa* and *C. roxburghii* were included in series *Obolospermae* and *C. grandis* to series *Grandes*. *Senna surratensis* in series Peiranisia while *Senna alata* to series Interglandulosae.

#### GC-MS metabolic profiling analysis

The metabolic profiling pattern of the tested plants was analyzed as metabolic marker for confirming the traditional taxonomical features and molecular DNA barcoding analysis [47]. Gas chromatography-mass spectrometry has been established as a key technological tool for metabolic profiling and taxonomical tools to confirm the traditional taxonomical features. The GC-MS metabolic profiling has been used frequently for taxonomic purposes 129 species belonging to 29 genera of the Convolvulaceae [88], six species of *Salvia* (Lamiaceae) [89], three species of the tree-fern *Cyathea* (Cyatheaceae) [90]. Eleven species of *Solanum* (Solanaceae) [91] *Centaurea galicicae* and *C. tomorosii* (Asteraceae) [92] and also for 14 species of that family [93]. GC-MS has immensely contributed to the detection of bioactive constituents from plants which might be very useful for drug research and discovery [30, 94].

**Fig. 5:**
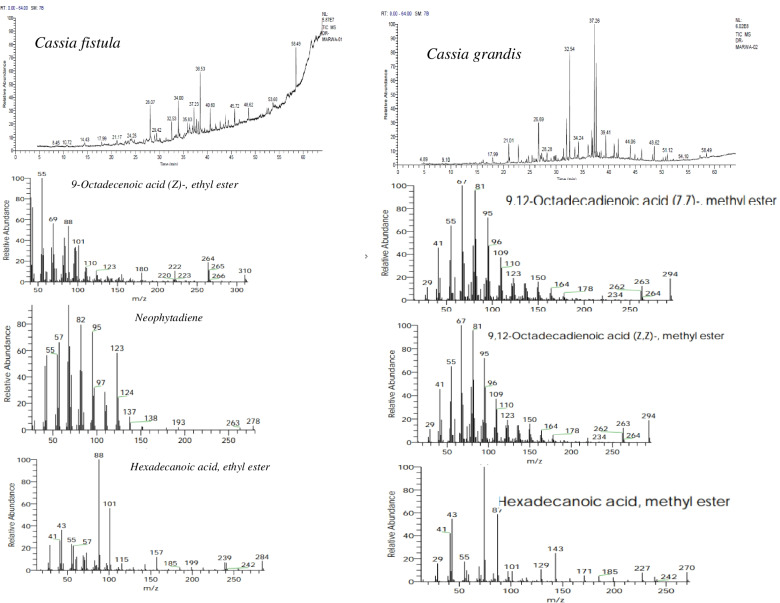
GC-MS chromatogram of methanol leaf extract of *Cassia fistula* and *Cassia grandis*

**Fig. 6:**
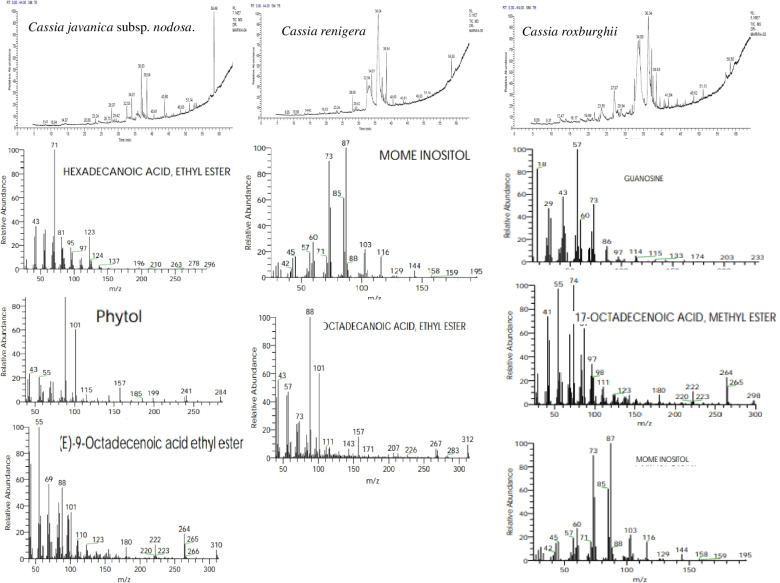
.

**Fig. 7:**
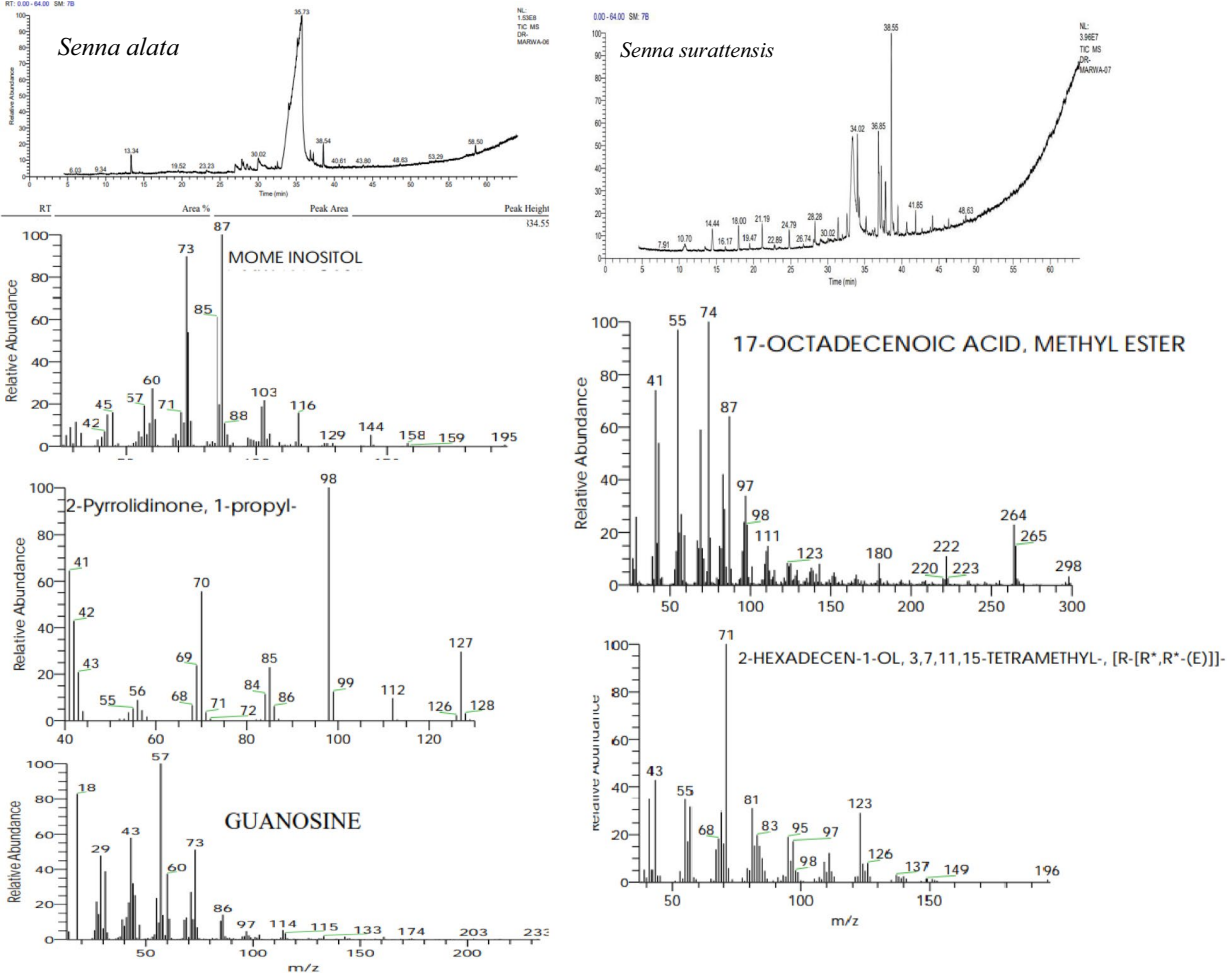
GC-MS chromatogram of methanol leaf extract of *Senna alata*, and *Senna surattensis*

An extensive survey of literature elucidated that there is no evidence for the utility of GC-MS screening of phytochemicals has been generated for the taxonomic investigation of genus *Cassia* from Egypt or anywhere else. Conversely, an immense phytochemical interest using GC-MS has been paid on *Cassia* sens. Lat. (including species of *Senna*) as a result of their excellent medicinal values [24, 67]. From the GC-MS profile (Table 9), 23 metabolic compounds were identified in the methanol extracts of leaves of the taxa, with obvious fluctuation on their concentrations, as revealed from the area of the peaks of chromatograms of GC-MS. The identified compounds of the studied taxa with their retention times, molecular formula, molecular weight, chemical class and concentration were represented. The GC-MS chromatograms were shown in Figs. 5, 6 and 7. The identified compounds were assigned to various chemical classes such as organosiloxane, esters, fatty acid esters, fatty acids and alcohols, hydrocarbons, phenolic compounds, carboxylic sugar and fat-soluble Vitamin E. The first compound identified, in the leaf extracts, was Cyclooctasiloxane, hexadecamethyl at a retention time 17.97 min in *Cassia fistula* and *C. grandis*, while Vitamin E was the last compound at the retention time 58.49 min in all the taxa investigated except *S. surtattensis*. The compounds Neophytadiene, Hexadecanoic acid, ethyl ester, 16-Octadecenoic acid, methyl ester, 9-Octadecenoic acid, ethyl ester and Vitamin E was highly dominant in *C. fistula*. While, the compounds 2,4-Di-tert-butylphenol, 1-Nonadecene, Hexadecanoic acid, methyl ester, 9-Octadecenoic acid, methyl ester, 9,12-Octadecadienoic acid (Z,Z), methyl ester were the most frequent in *C. grandis.* Vitamin E and phytol were the most dominant metabolites in *C. javanica* subsp. *nodosa* followed by 9-Octadecenoic acid, ethyl ester and Hexadecanoic acid. Myo-Inositol was the most frequent metabolite in *Cassia renigera* (46.5%) followed *C. roxburghii* (19.8%). Remarkably, the biological and chemical identities of the metabolites of the genera *Cassia* was distinctly different from the genera of *Senna*. The most dominant compounds of *S. alata and S. surratensis* were different from that of *Cassia* sp., ensuring the metabolic difference of gene expression pattern on both genera. Out of the 23 phytochemical compounds identified, four compounds detected at different retention times and with varied concentrations displayed a consistent occurrence among the taxa investigated. These common compounds comprised Hexadecanoic acid, methyl ester; Hexadecanoic acid, ethyl ester; 9-Octadecenoic acid (Z)-ethyl ester and Vitamin E (Table 9). Several of the scored phytochemical compounds were demonstrated as unique chemical traits for individual species; such for instances *C. fistula, C. grandis*, *C. javanica* subsp. *nodosa* and *C. roxburghii.* Furthermore, the absence of 16-Octadecenoic acid, methyl ester and Oleic acid at the retention time 37.2 and 40.6 min, respectively, could be characteristic for *C. grandis*, while the absence of 1,2-benzenedicarboxylic acid, bis(2-ethylhexyl) ester could be diagnostic for *C. renigera*, *S. alata* and *S. sutattensis* (Table 9).

**Table 9:** The identified phytocompounds in the methanolic extracts of leaves of the taxa studied of *Cassia* and *Senna*

RT	Phytocompounds	MF	MW	Chemical class	Area %						
					sp_**1**_	sp_**2**_	sp_**3**_	sp_**4**_	sp_**5**_	Sp6	Sp7
17.97	Cyclooctasiloxane, hexadecamethyl-	C16H48O8Si8	592	Organosiloxane	0.60	1.23	–	–	–	–	–
21.01	1-Hexadecanol	C16H34O	242	Cetyl alcohol (16 C fatty alcohol)	–	2.06	–	–	–	–	–
22.85	2,4-Di-tert-butylphenol	C14H22O	206	Alkyl benzene	–	2.57	–	–	–	–	–
23.24	2,6-Diflurobenzoic acid, tridec-2-ynyl ester	C20H26F2O2	336	Ester	–	–	1.55	–	–	–	–
23.55	Phenol, 2-propyl-	C9H12O	136	Propyl phenol	–	–	–	–	1.70	–	–
26.69	1-Nonadecene	C19H38	266	Un-branched 19 C alkene	–	5.48	–	–	–	–	–
27.07	Guanosine	C10H13N5O5	283	Purine nucleoside	–	–	–	–	6.38	**2.60**	–
28.08	Neophytadiene	C20H38	278	Diene Hydrocarbon	4.63	–	3.87	3.16	–	–	–
28.94	Tetradecanoic acid	C14H28O2	228	Fatty acid	–	–	–	–	1.37	–	–
32.54	Hexadecanoic acid, methyl ester	C17H34O2	270	Fatty acid ester	2.44	12.45	3.93	3.46	2.44	–	–
34.01	Hexadecanoic acid, ethyl ester	C18H36O2	284	Fatty acid ester	4.59	0.63	5.13	3.67	4.14	5.5	5.7
36.04	Myo-Inositol, 2-C-methyl-	C7H14O6	194	Carbocyclic sugar	–	–	–	46.5	19.86	49.59	–
36.83	Phytol	C20H40O	296	Acyclic diterpene alcohol	–	–	11.99	–	–	**3.20**	**10.31**
37.23	16-Octadecenoic acid, methyl ester	C19H36O2	296	Fatty acid ester	3.07	–	3.96	3.61	4.06	**2.24**	**4.74**
37.26	9-Octadecenoic acid, methyl ester (E)-	C19H36O2	296	Fatty acid ester	–	15.81	–	–	–	–	–
37.61	9,12-Octadecadienoic acid (Z,Z)-, methyl ester	C19H34O2	294	Fatty acid ester	–	10.06	–	–	–	–	–
38.54	9-Octadecenoic acid (Z)-, ethyl ester	C20H38O2	310	Fatty acid ester	7.13	1.15	9.30	8.63	3.58	**5.25**	**0.61**
40.60	Oleic acid	C18H34O2	282	Fatty acid	2.93	–	1.43	1.25	0.82	–	–
43.80	Meadowlactone	C20H38O2	310	Delta-lactone	–	–	4.47	–	–	–	–
45.72	9-Octadecenoic acid (Z)-, 2,3-dihydroxypropyl ester	C21H40O4	356	Monoacylglycerol	2.08	–	–	–	–	–	–
48.62	1,2-Benzenedicarboxylic acid, bis(2-ethylhexyl) ester	C24H38O4	390	Aromatic dicarboxylic acid ester (Ester)	1.44	1.75	1.00	–	0.71	–	–
53.68	Docosanoic acid, 1,2,3-propanetriyl ester	C69H134O6	1058	Fatty acid ester	1.38	–	–	–	–	–	**0.38**
58.49	Vitamin E	C29H50O2	430	Fat-soluble vitamin	5.11	0.64	18.67	3.57	1.39	**2.04**	**0.57**

*-* Absent, *MF* Molecular formula, *MW* Molecular weight, *sp*_*1*_*Cassia fistula*, *sp*_*2*_*Cassia grandis*, *sp*_*3*_*Cassia javanica* subsp. *nodosa*, *sp*_*4*_*Cassia renigera*, *sp*_*5*_*Cassia roxburghii*, *Senna alata, sp6 Senna surattensis*

The present study comparatively explores the taxonomic framework, phytochemical constituents of leaves of six species of *Cassia* and *Senna* and one subspecies of genus *Cassia* from Egypt via GC-MS screening for use as chemical markers for classification of plants, [91, 93], for the therapeutic agents, [30]. The chemical information of plants can provide new taxonomic diagnostic characters that help to improve classification of plants [95]. Hexadecanoic acid, methyl ester; Hexadecanoic acid, ethyl ester; 9-Octadecenoic acid (Z)-ethyl ester and Vitamin E displayed a consistent occurrence among the taxa investigated. Thus, they can be designated, here, as the chemotaxonomic markers for the taxa investigated of *Cassia* and *Senna* at the genus level. Besides, several of the identified were assigned as exclusive diagnostic chemical traits for individual taxa, for example, 9-octadecenoic acid, 2,3-dihydroxypropyl ester and Docosanoic acid, 1,2,3-propanetriyl ester for *C. fistula*, while 1-Hexadecanol, 2,4-Di-tert-butylphenol, 1-Nonadecene and others are diagnostic for *Cassia grandis*. The lack of certain compounds and presence of other compound at the same retention times may be considered as chemotaxonomic guides for some species. For example, the absence of 16-Octadecenoic acid, methyl ester and oleic acid may be characteristic for *Cassia grandis*, while the absence of 1,2-Benzenedicarboxylic acid, bis(2-ethylhexyl) ester may be diagnostic for *C. renigera*. Based on vegetative and reproductive characteristics, *C. fistula* was assigned to series *Cassia* and *C. grandis* to series *Grandes*, while *C. javanica* and *C. roxburghii* were included in series *Obolospermae.* Hence, the taxa studied of genus *Cassia* may find their sound to be utilized in identification of potential lead compounds very useful for discovery of novel pharmaceuticals. For instance, Heaxdecanoic acid, methyl ester was reported to exhibit anti-inflammatory and antifibrotic activities [96]. 9-Octadecenoic acid (Z)-,2,3-dihydroxypropyl ester was regarded as a magic lipid regarding its diverse application in pharmaceuticals, cosmetics, food and protein crystallization Powder- [97]. They added that this compound is known for its surfactant and emulsifying properties. Besides, its use as a drug delivery enhancer was documented [98]. Fatty acids are carboxylic acids with an aliphatic chain which are either saturated or unsaturated [99]. Monounsaturated and polyunsaturated fatty acids have been utilized to lower the risk of heart disease and also to enhance the immune system [100]. Herein, Oleic acid; is one of the unsaturated fatty acids, has been reported to exhibit various bioactivities such as anti-inflammatory, cancer preventive, hypochloestrolemic and dermatitigenic [101]. The compound 1,2-Benzenedicarboxylicacid, bis(2-ethylhexyl) ester was isolated from twigs of the dicot flowering plant *Thevetia peruviana* as a potential biomarker [102]. They added that this compound was proved to be a strong immunomodulatory B-cell stimulant. Moreover, this compound revealed positive anticancer activity on PC3, MCF and other cancer cell lines. Phytol is an acyclic diterpene alcohol which is a precursor for vitamins E and K1 [66] It results from the hydrolysis of chlorophyll and was found to be effective at different stages of arthritis [103]. Moreover, phytol was found to have antibacterial activities against *Staphylococcus aureus* [104]. Neophytadiene was reported as presenting antimicrobial and anti-inflammatory activities [105]. Vitamin E is a fat-soluble compound that functions as antioxidant in human body system [106]. 2,4-Di-tert-butyl phenol is a lipophilic phenol produced by various groups of organisms as a common toxic secondary metabolite [107].

## Conclusions

Few taxonomical studies on the genus *Cassia* and *Senna,* were published regard to the biological identity of these plants as repertoire to various bioactive compound. Thus, the objective of the current was to revise and authenticate the phylogenetic relationship between studied taxa of the species of *Cassia* and *Senna* in Egypt using the recent tools of ITS barcoding, RAPD analysis and metabolic profiling, in comparing to the traditional taxonomical features. The taxonomical description of the studied taxa was confirmed from the molecular analysis of ITS sequences and RAPD analysis. Thus, from the molecular analysis, two clades were clearly separated into Clade I of *Cassia* and Clade II of *Senna*. The cluster I represented by *C. fistula*, C. *renigera*, *C. roxburghii*, and *C. javanica* sub *nodosa,* and the cluster II represented by *S. alata* and *S. surattensis.* The morphological, molecular traits of the studied plants were authenticated from the metabolic profiling by GC-MS analysis. The identified compounds were potentially useful for both the taxonomic purpose and pharmaceutical applications. The study highlighted the pharmaceutical significance of several of the identified phytochemicals. From the taxonomical view, the genetic links between members of the Cassiineae, namely the *Cassia*, *Senna* genus are solved and morphological observations support. Conclusively, the traditional morphological features, molecular barcoding using ITS sequences, RAPD analysis and metabolic traits by GC-MS analysis, authenticates the taxonomical diversity of the genus *Cassia* and *Senna*.

## Supplementary Information

**Additional file 1: Table S1.** List of 28 morphological character and their state in the seven studied taxa of *Cassia* and *Senna*.

**Additional file 2.**

## Data Availability

The datasets used and analyzed during the current study available from the corresponding author on reasonable request. The accession numbers of the deposited ITS sequences were listed on Table 4.
