# Supplementary material for: Molecular and metabolic traits of some Egyptian species of Cassia L. and Senna Mill (Fabaceae-Caesalpinioideae)
Source: BMC Plant Biol. 2022 Apr 20;22:205. doi: 10.1186/s12870-022-03543-7 (PMC9020050; doi:10.1186/s12870-022-03543-7)
Supplement: Supplementary file 1 — Additional file 1: Table S1. List of 28 morphological character and their state in the seven studied taxa of Cassia and Senna. [file 12870_2022_3543_MOESM1_ESM.docx]

**Table S1. List of 28 morphological character and their state in the seven studied taxa of *Cassia* and *Senna***

| Character States | Characters | No. |
| --- | --- | --- |
| Perinnal(1),annul(2) | Life span | 1 |
| Tree(1),),Shrub(2), Small tree(3) | Life form | 2 |
| Glabrous (1), Pubescent(2) | stem surfces | 3 |
| Deciduous (1), semidecidous (2),evergreen (3) | Leaf duration | 4 |
| with 8-14 pairs many (1)16-20 numerous(2) | Leaflet pairs in numbers | 5 |
| Ovate(1), oblong(2),obovate to oblong(3) Lanceolate (4) | Leaflet shape | 6 |
| Entire (1), not entire(2) | Leaflet margin | 7 |
| Acute (1), obtuse (2),Acuminate,(3) ,Obtuse rounded (4) | Leaflet apex | 8 |
| Obtuse(1), Oblique(2), Rounded(3) | Leaflet base | 9 |
| Glabrous(1), puberulent (2) | Leaflet adaxial surface | 10 |
| glabrous(1) Puberulent(2), tomentose(3 | Leaflt abaxial surface | 11 |
| ≥15(1),≥12(2,)≥10(3),≥5(4) | Leaflet lenght | 12 |
| ≤8 (1),≤ 2 (2),6 ≤,(3) | Leaflet width | 13 |
| Absent(1),present(2) | Petiolar glands | 14 |
| Persistent(1), Cauducous(2) | Stipule | 15 |
| deltoid to ovate (1), kidney (2), Oblong(3), linear to lanceolate (4), Triangular (5), liner(6) | Stipules shape | 16 |
| Ovate(1), leafy(2), Linear(3), oblong to broadly ovate(4), obliquely cordate(5), linear to lanceolate(6) | Bract shape | 17 |
| Ovate(1),oblong(2),obtuse(3) | Sepals shape: | 18 |
| Yellowish to green(1), redish(2),green(3),yellow(4), | Sepals colour | 19 |
| Obovate(1), Ovate Oblong(2), ovate-orbicular(3),ovate(4) | Petals shape | 20 |
| Yellow(1),pink(2) | Petals colour | 21 |
| Straight(1), Carved slighty(2) | Pod curvature | 22 |
| Rouded (1),acute(2),acuminate(3) | Pod apex | 23 |
| Dark brown(1),brown(2),black(3) | Pod colour | 24 |
| Glabrous(1), hairy(2) | Pod Texture d | 25 |
| Indehiscent(1), dehiscent(2) | Dehiscence of Pod | 26 |
| obovate-elliptic(1) elliptic(2), deltoid(3),oval(4),obovate oblong(5) | Seed shape | 27 |
| light brown(1),brown(2),black(3),Darkbrown (4) | Seed color | 28 |
