# Supplementary figures and images for: Molecular and metabolic traits of some Egyptian species of Cassia L. and Senna Mill (Fabaceae-Caesalpinioideae)
Source: BMC Plant Biol. 2022 Apr 20;22:205. doi: 10.1186/s12870-022-03543-7 (PMC9020050; doi:10.1186/s12870-022-03543-7)

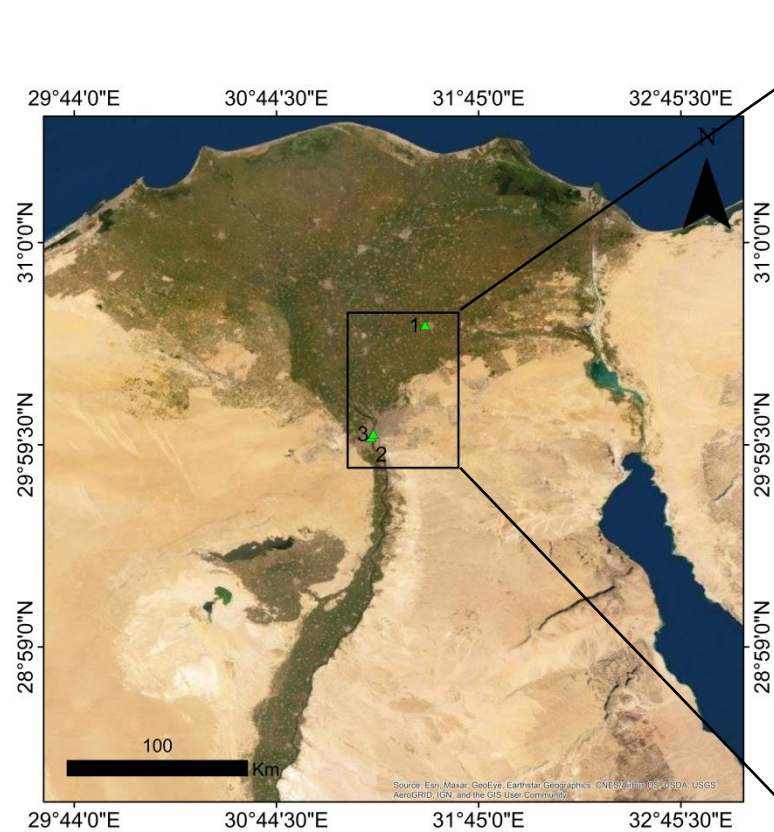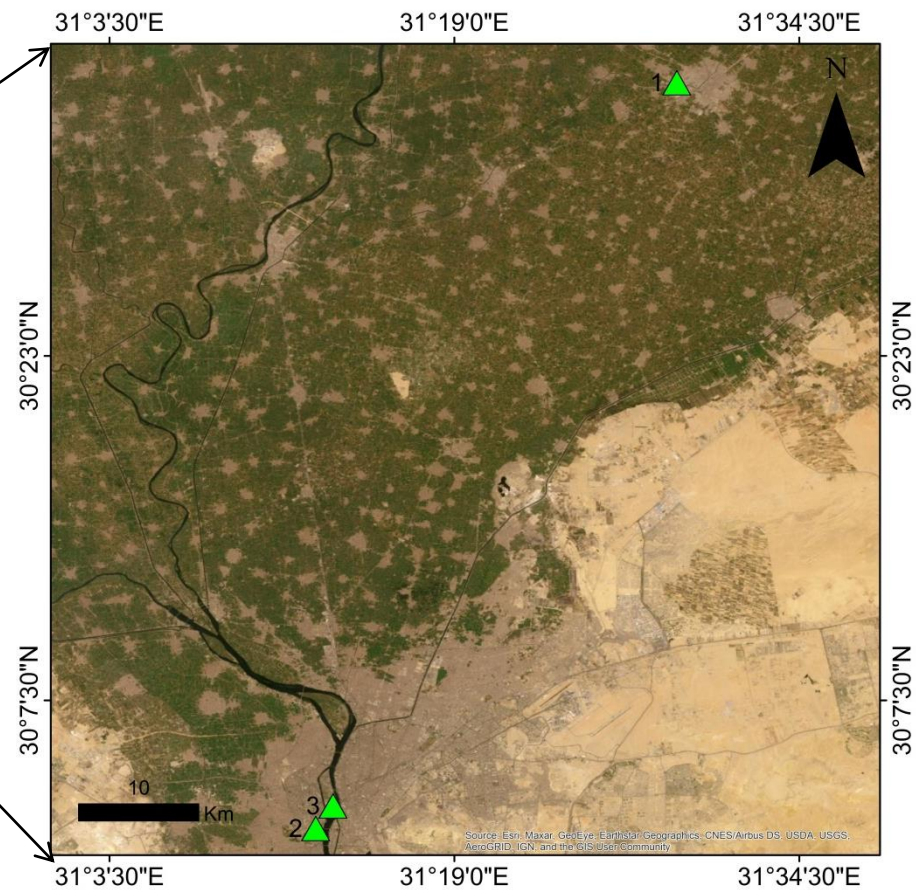

Supplement: Supplementary file 2 — Additional file 2. [file 12870_2022_3543_MOESM2_ESM.pdf]
